# The association of appointment type in healthcare with patient reported experience measures: a population-based survey study

**DOI:** 10.1186/s12913-025-12869-5

**Published:** 2025-05-20

**Authors:** Tarja Heponiemi, Emma Kainiemi, Lotta Virtanen, Tuulikki Vehko, Anna-Mari Aalto

**Affiliations:** Finnish Institute for Health and Welfare, P.O. Box 30, Helsinki, 00271 Finland

**Keywords:** Teleconsultations, Patient-reported measures (PREMs), Time allocation, Patient participation, Responsiveness to needs, Health care

## Abstract

**Background:**

Poor access to healthcare and long waiting times are severe challenges in many countries and therefore countries have increasingly adopted teleconsultations such as video, messaging, and phone calls. Patient-reported experience measures (PREMs) assess the quality of care based on patients’ insights covering topics like the quality of communication, patient participation, and adequacy of time allocation of the appointment. The present study examined whether the type of appointment (in-person, by phone call or via digital services), service sector, and encountered health professional were associated with patients’ experience of appointment quality in healthcare.

**Methods:**

The data from the population-based cross-sectional Healthy Finland Survey conducted from September 2022 to February 2023 including 22 665 respondents (53% women) were used. Complex samples logistic regression analyses were used to examine the associations of the independent variables (type of appointment, the service sector and encountered healthcare professional) with PREMs (sufficient time allocation, opportunity to ask questions, active participation, and responsiveness to needs) adjusted for self-rated health, age, sex, and urbanization status.

**Results:**

Those whose appointment was conducted by phone call had greater odds of not agreeing that enough time was allocated (OR = 1.57, 95%CI = 1.36–1.81), opportunity to ask was offered (OR = 1.28, 95%CI = 1.10–1.50), active participation possibilities were given (OR = 1.33, 95%CI = 1.15-1.54-), and their needs were met (OR = 1.39, 95%CI = 1.20–1.60) compared to in-person appointments. Those whose appointment was conducted by digital services had greater odds of not agreeing that enough time was allocated (OR = 1.51, 95%CI = 1.21–1.88) and opportunity to ask was offered (OR = 1.38, 95%CI = 1.07–1.78) compared to in-person appointments. Moreover, respondents had greater odds of disagreeing with PREM statements in health centers and when encountering physicians compared to their counterparts.

**Conclusions:**

It seems that teleconsultations do not allocate enough time, offer opportunity to ask, give possibility to active participation, and meet patients’ needs similarly as in-person appointments. Especially appointments conducted by phone call differed negatively from in-person visits regarding all these aspects. Our findings should be kept in mind when planning and developing teleconsultations. Teleconsultations may be a good option in many cases, but not for all patients or for all situations. Moreover, physicians and health centers should adopt means to improve time allocation, patient participation, and responsiveness to patients’ needs.

## Background

Poor access and long waiting times to healthcare are severe challenges in many countries [1]. One solution that countries have adopted to improve the situation is digitalization of healthcare services such as increased use of teleconsultations [2–6]. Teleconsultations are remote appointments between patients and healthcare professionals using digital communication methods such as video, messaging, or phone calls. Teleconsultations may pose challenges to the communication related to technical issues, difficulties in developing relationship, lack of non-verbal communication and physical examination, language barriers, spatial issues, and difficulties in assessing patients’ health literacy [7, 8]. A previous study found that the majority of patients preferred in-person visits over teleconsultations due to perceptions of more accurate diagnoses, better examinations, and improved treatment of their health conditions [9]. However, increased convenience, time savings and the possibility for a quick response in case of urgent needs have been identified as benefits by patients [8, 10]. Thus, it can be assumed that patients’ experiences of their appointment with healthcare professional may vary according to the type of the appointment, whether in-person, by phone call or by digital services.

It is expected that in-person appointments offer patients personalized, face-to-face interactions, which can enhance the accuracy of diagnoses and the effectiveness of treatments, given that in-person appointments allow performance of thorough physical examinations, obtain diagnostic tests on-site, and gain a thorough understanding of the patient’s condition [9]. Instead, phone call appointments offer a more accessible and flexible option for patients, especially for those in remote areas or with mobility issues. Moreover, digital appointments are expected to offer convenience, promptness, and enhanced communication [11].

One fundamental element of high-quality healthcare and patient-centered care is the use of patient-reported experience measures (PREMs), which assess the quality of care based on patients’ insights. The Organisation for Economic Co-operation and Development (OECD) emphasizes the need to understand how healthcare impacts patients’ lives, noting that health outcomes alone provide only a limited perspective on a health system’s overall performance [12]. Thus, measuring PREMs offer an opportunity to improve care and optimize healthcare performance [12–14]. The PREMs typically cover topics like the quality of communication between patient and healthcare provider and the possibility of patient to participate in care-related decision-making. The adequacy of time allocated to the patient during their appointment is one important PREM related to the quality of communication which often can be experienced as inadequate by patients.

A patient’s ability to participate in decision-making is crucial for ensuring high-quality care and achieving positive healthcare outcomes. Participation can involve a collaborative relationship, the exchange of information, shared knowledge and power, as well as patients’ active engagement in treatment planning and decision-making [15, 16]. Shared decision-making is characterized by an ongoing dialogue between the healthcare provider and the patient, during which the patient gains important knowledge for making informed decisions [15]. Positive experiences of shared decision-making have been found to be associated with lower patient distress and care burden and higher perceived care quality and self-management [17, 18]. Ideally, professionals should provide sufficient information to patients; however, patients often express dissatisfaction with the amount and quality of information received [19]. Research suggests that patients may struggle to ask clarifying questions unless explicitly encouraged [20, 21]. Moreover, responsiveness to patients’ needs is a key aspect of healthcare [22], but previous study has found that patients’ needs may be unrecognized, and the priority of professionals may not reflect patients’ own priorities [23].

With the rapid increase of teleconsultations, it is important to gain a deeper understanding of how patients perceive these appointments in addressing their needs and whether sufficient time and opportunities for participation have been provided. This study aimed to examine whether the type of appointment (in-person, by phone call or by digital services), service sector and encountered healthcare professional adjusted for patient background variables were associated with patients’ experiences of appointment quality in healthcare (including sufficient time allocation, opportunity to ask questions, active participation, and responsiveness to needs) among Finnish population. Finland offers a good context for examining teleconsultations given that Finland is amongst the forerunners in digitalization and the adoption rate of digital healthcare services in the country is high [24]. Moreover, the Finnish Government highlights the need to promote the use of digital healthcare services, prioritizing them as a primary mode of service delivery whenever suitable and when they respond to patients’ needs [25].

## Methods

### Sample

The Healthy Finland Survey was a cross-sectional population-based questionnaire survey conducted from September 2022 to February 2023. The Healthy Finland surveys include regularly conducted large national surveys providing reliable and up-to-date information on the health, well-being and service use of adults living in Finland [26]. The sample was randomly selected and stratified by wellbeing service counties (21 counties and Helsinki capital city as an independent region). From each county, 2 800 persons (2 000 persons aged 20–74 years and 800 aged 75 or older) were drawn from the population registry maintained by the Digital and Population Data Service Agency (DVV). The questionnaire was tailored according to age group (20–54 years / 55–74 years / 75 or older). The measures used in the present study were included to questionnaires among all age groups. The questionnaire was available in Finnish and Swedish (official languages in Finland), Russian, and English. It was possible to respond either online or by paper. The two younger (those between 20 and 74 years) age groups received a postal invitation with address to the online questionnaire. The oldest age group received a paper questionnaire with the invitation to respond. For those who had not responded, the recruitment protocol included two text messages (SMS) and three postal reminders, one of which also included the paper questionnaire. The language of the paper questionnaire was based according to the communication language of the person received from DVV. Upon request, a paper questionnaire could be provided in the preferred language of the respondent from the four available language options.

A total of 65 986 persons were invited to the study of whom 28 154 responded (response rate 46.3%). The respondents were asked whether they had used healthcare services in the past 12 months. Only those respondents who answered that they had used healthcare services in the past 12 months (*N* = 22 665, 53% women) were included to the present study.

An Inverse Probability Weighting (IPW) correction based on register data on age, sex, marital status, education level, region of residence, native language, and possible hospitalizations was used to address the potential bias. Previous studies have shown the suitability of this method for correcting possible non-response bias among the Finnish population [27].

### Ethical issues

Participation in the study was completely voluntary, participants’ anonymity was respected, and the participants were provided with an opportunity to withdraw from the study. The study received an approval from the Institutional Review Board of the Finnish Institute for Health and Welfare (THL/72/6.02.01/2022).

### Measurements

#### Dependent variables

Respondents’ experiences about their most recent appointment with a healthcare professional was assessed using four questions. Three of these questions focusing on communication and shared decision-making were derived from the OECD’s PREMs [12, 28]. To better align with the Finnish healthcare system, which assigns a lot of responsibilities to nurses, the original OECD questions were modified to include other healthcare professionals in addition to physicians. To ensure the quality of the modified Finnish questions, forward-back translation method was used.

In addition, we included a more general question specifically designed for this study, addressing whether the patient’s needs were met during the appointment.

The respondents were asked: “The following questions relate to the interaction with the professional you met (doctor, nurse or other healthcare professional) during your most recent appointment:

Was enough time spent with you during your appointment? (named as *Sufficient time allocation)*

Could you ask questions or express concerns about the recommended care? (*Opportunity to ask*)

Did you get to participate in the decisions concerning your care as much as you wanted to? (*Active participation*)

Did the service meet your need? (*Responsiveness to needs)*

Response options were (a) absolutely yes, (b) to some extent, (c) not really, (d) absolutely not, and (e) cannot say. Those who answered cannot say, were marked as missing (n ranged between 305 and 1235). For the analysis, the variable was coded as 0 = fully agree (response option absolutely yes) and 1 = not fully agree (response options b, c, and d).

#### Independent variables

Three variables described the most recent appointment in healthcare by asking: ”The following questions relate to your most recent dealings with a healthcare professional.” *The appointment type* of the most recent appointment was assessed by asking: “How did you manage your affairs?” with response options (a) visiting in-person (at the professional’s reception), (b) remotely by phone and (c) remotely by digital services (via video or chat). *The service sector* of the most recent appointment with a healthcare professional was asked with response options: health center, private medical clinic, occupational health, hospital outpatient clinic, and other. *The encountered healthcare professional* was asked as (1) a registered nurse or public health nurse, (2) a general practitioner or medical specialist, and (3) another health professional.

#### Adjustment variables

Self-rated health, age, sex, and urbanization status were used as adjustment variables. The respondent’s self-rated *health* was measured by a widely used question from previous national health surveys in Finland: ‘How is your present state of health?’, with the response options (a) good, (b) rather good, (c) moderately good, (d) rather poor and (e) poor. This wording differs from the widely used European Health Interview survey question (https://ec.europa.eu/eurostat/statistics-explained/index.php?title=Glossary:Minimum_European_Health_Module_(MEHM)) but was chosen for this study to follow national time trends [29, 30]. The measure was coded as 1 = good health (response options a and b) and 2 = poor health (response options c–e). *Age* (continuous), *sex* (female, male), *and urbanization status* of the respondent (urban, semi-urban, and rural) were obtained from the National Population Register.

### Statistical analysis

Complex samples logistic regression analyses were used to examine the associations of the independent variables with dependent variables (each in separate analyses). The multivariable model for each dependent variable (sufficient time allocation, opportunity to ask, active participation, and responsiveness to needs) included appointment type, the service sector, encountered professional, self-rated health, age, sex, and urbanization status. The analyses were conducted using SPSS 29.0.2.0 statistical package. Methods suitable for weighted data were used: Complex Samples Logistic regression analyses and Complex Samples Descriptives/Frequencies for descriptive statistics. Some variables included missing data which were deleted from the analyses, thus n ranges between 17,464 and 18,312 in the statistical analyses. We assessed multicollinearity using the Variance Inflation Factor (VIF). The VIF values for all predictor variables ranged between 1.01 and 1.12, indicating minimal multicollinearity.

## Results

### Characteristics of the sample

Characteristics of the weighted study sample can be seen in Table 1. Majority of the respondents lived in urban areas. The most common encountered health professional was a physician, and the most common service sector was health center. Majority of the respondents had met the healthcare professional in-person, whereas appointments by phone call or by digital services were less common.

**Table 1:** Characteristics of the study sample

Variable	*n*	%
Sufficient time allocation		
Absolutely yes	14,275	65.0
To some extent	5847	26.6
Not really	1067	4.9
Absolutely not	366	1.7
Cannot say	415	1.9
Opportunity to ask		
Absolutely yes	16,416	74.9
To some extent	3931	17.9
Not really	830	3.8
Absolutely not	124	0.6
Cannot say	612	2.8
Active participation		
Absolutely yes	13,859	63.4
To some extent	4886	22.3
Not really	1533	7.0
Absolutely not	338	1.5
Cannot say	1256	5.7
Responsiveness to needs		
Absolutely yes	15,151	69.1
To some extent	5222	23.8
Not really	897	4.1
Absolutely not	338	1.5
Cannot say	305	1.4
Appointment type		
In person	18,161	84.6
Remotely by phone	2154	10.0
Remotely digitally	1162	5.4
Service sector		
Health centre	7514	35.7
Private medical clinic	3616	17.2
Occupational health	5824	27.7
Hospital outpatient clinic	3409	16.2
Other	663	3.2
Encountered health professional		
Nurse	4748	22.6
Physician	15,245	72.5
Other	1033	4.9
Self-rated health		
Good health	13,917	62.3
Average or poor health	8415	37.7
Sex		
Male	10,455	46.5
Female	12,010	53.5
Urbanization status		
Urban	16,413	73.1
Semi-urban	3386	15.1
Rural	2665	11.9
	Mean	SE
Age	52.3	0.16

The table presents characteristics of the weighted data

### Independent factors’ associations with PREMs

The results of the multivariable logistic regression analyses are presented in Table 2. Appointment type, the service sector, encountered professional, self-rated health, age, and sex were significantly associated with sufficient time allocation when controlled for all variables. Respondents whose appointments were conducted by phone call or by a digital service had greater odds of not fully agreeing that sufficient time was allocated to them during the appointment compared to those with in-person appointments. Compared to appointments organized by health centers, respondents in all other service sectors had lower odds of not fully agreeing that sufficient time was allocated to them. Those who met a physician at their recent appointment had greater odds of not fully agreeing about sufficient time compared to those who met a nurse.

**Table 2:** Logistic regression analyses for not fully agreeing that most recent appointment offered sufficient time allocation, opportunity to ask, active participation and responsiveness to needs. Odds ratios and their 95 per cent confidence intervals

Variable	Sufficient time allocation			Opportunity to ask			Active participation			Responsiveness to needs		
	OR	95% CI	*p*	OR	95% CI	*p*	OR	95% CI	*p*	OR	95% CI	*p*
Appointment type			< 0.001			< 0.001			< 0.001			< 0.001
In person	1			1			1			1		
Remotely by phone	1.57	1.36–1.81		1.28	1.10–1.50		1.33	1.15-1.54-		1.39	1.20–1.60	
Remotely digitally	1.51	1.21–1.88		1.38	1.07–1.78		1.07	0.83–1.38		1.15	0.92–1.43	
Service sector			< 0.001			< 0.001			< 0.001			< 0.001
Health centre	1			1			1			1		
Private medical clinic	0.49	0.42–0.56		0.47	0.40–0.56		0.43	0.37–0.50		0.38	0.32–0.44	
Occupational health	0.60	0.53–0.68		0.55	0.48–0.64		0.54	0.47–0.61		0.57	0.50–0.65	
Hospital outpatient clinic	0.79	0.70–0.90		0.74	0.64–0.86		0.84	0.74–0.95		0.62	0.54–0.71	
Other	0.63	0.46–0.87		0.56	0.39–0.80		0.52	0.37–0.73		0.77	0.56–1.04	
Encountered professional			< 0.001			0.004			< 0.001			0.021
Nurse	1			1			1			1		
Physician	1.44	1.28–1.62		1.16	1.02–1.32		1.26	1.12–1.42		1.18	1.05–1.33	
Other	1.16	0.90–1.47		0.80	0.60–1.06		1.15	0.90–1.48		1.20	0.95–1.52	
Self-rated health			< 0.001			< 0.001			< 0.001			< 0.001
Good health	1			1			1			1		
Average or poor health	1.97	1.80–2.16		1.84	1.65–2.05		1.83	1.67–2.01		2.18	1.98–2.40	
Age^1^	0.99	0.99-1.00	< 0.001	0.99	0.99-1.00	< 0.001	1.00	0.99-1.00	0.003	0.99	0.99–0.99	< 0.001
Sex			0.011			0.746			0.002			0.425
Male	1			1			1			1		
Female	0.89	0.81–0.97		0.98	0.88–1.10		0.86	0.78–0.95		0.96	0.87–1.06	
Urbanization status			0.330			0.044			< 0.001			0.027
Urban	1			1			1			1		
Semi-urban	0.93	0.82–1.05		0.89	0.77–1.02		0.81	0.72–0.92		0.88	0.77-1.00	
Rural	0.93	0.83–1.05		0.86	0.75–0.98		0.82	0.72–0.93		0.87	0.76–0.98	

*OR* Odds ratio, *CI* Confidence interval

^1^Age was used as continuous variable in the analyses, thus odds ratio reflects the change for one unit (year) change

Appointment type, the service sector, encountered professional, self-rated health, age and urbanization status were significantly associated with opportunity to ask when all variables were controlled for. On average, those whose appointment type was by phone call or digital service had greater odds of not fully agreeing that they had opportunity to ask during the appointment compared to those whose appointment type was in-person. Compared to respondents whose appointment was organized by health centers, those who used other service sectors had lower odds of not fully agreeing that they had opportunity to ask during the appointment. Those who had their recent appointment with physician had greater odds of not fully agreeing compared to those whose appointment was handled by nurse.

All the examined independent variables were significantly associated with active participation when all the variables were controlled for. Respondents whose appointment type was by phone call had greater odds of not fully agreeing that they were able to participate to decision-making during the appointment compared to those whose appointment type was in-person. Respondents in all other service sectors had lower odds of not fully agreeing that they were able to participate to decision-making during the appointment compared to respondents whose service was provided by health centers. Those who had their recent appointment with physician had greater odds of not fully agreeing compared to those whose appointment was handled by a nurse.

Appointment type, the service sector, encountered professional, self-rated health, age, and urbanization status were significantly associated with responsiveness to needs, controlling for all the variables. Respondents whose appointment type was by phone call had greater odds of not fully agreeing that their needs were met during the appointment compared to those whose appointment type was in-person. Respondents in all other service sectors had lower odds of not fully agreeing that their needs were met during the appointment compared to the respondents whose service was organised by health centers. Those who had their recent appointment with physician had greater odds of not fully agreeing compared to those whose appointment was handled by a nurse.

## Discussion

The present study examined whether the type of the appointment (in-person, by phone call or by digital services) was associated with patients’ experiences of their recent healthcare appointment in Finland. Based on our findings, it seems that phone appointments, in particular, may face challenges in patients’ perceptions of sufficient time, providing opportunities for patients to ask questions, encouraging active participation, and meeting patients’ needs. For digital appointments, the primary difficulties arose in ensuring sufficient time allocation and providing opportunities for patients to ask questions. Moreover, the service sector and the healthcare professional encountered during the recent appointment, as well as patient’s background characteristics were associated with patients’ experiences of appointment quality.

Our results give further support to previous research indicating that teleconsultations are not experienced to fulfill important aspects of high-quality care to the same extent as in-person appointments. For example, it has been found that the length of teleconsultations is shorter than that of in-person visits on average [31]. Moreover, previous findings show that the majority of patients preferred in-person visits over teleconsultations [9]. Teleconsultation appointments have been considered less ‘information rich’ than in-person appointments [31]. However, there are also studies where patients have more positive perceptions regarding their satisfaction with communication, usefulness, and quality of teleconsultations [32, 33].

According to our results, patients might experience difficulties in phone appointments related to their participation in decision-making and the responsiveness of care to their needs. This same experience does not apply for digital services. A previous study found that video consultations offered better opportunities to building relationship than phone calls, whereas there were no differences regarding consultation length, content, and quality [31]. It is possible that video appointments may provide better communication opportunities compared to phone calls, which are restricted to verbal communication [34]. Indeed, the loss of valuable non-verbal communication has been identified as a key disadvantage of teleconsultations [8]. Many previous studies on teleconsultations have not focused on comparing appointments conducted by phone calls and digital services. However, according to our results, it is important to examine them separately given that phone appointments may be regarded differently and perhaps more critically. Comparing phone appointments and video consultations might provide more specific information about whether video consultations are perceived as better, for example, in terms of communication, than phone calls. Moreover, our study did not separate video consultations and chat consultations, thus future studies should also compare how patients experience them.

Teleconsultation may be a good option for many patients such as those with chronic conditions who need regular follow-ups and those with long or difficult (e.g., due to disabilities) travel to healthcare [34]. Instead, they are not a good alternative for those who need a thorough physical examination or have communication challenges by phone or video [34]. Similarly, it has been concluded that video consultation is suitable for simple problems not requiring physical examination [31].

According to our results it seems that patients have more negative experiences related to their appointment in health centers or when having encountered physicians compared to their counterparts. Thus, some means should be conducted to improve time allocation, patient participation, and responsiveness to patients’ needs of appointments conducted in health centers or by physicians. It is worrying that previous study shows that physicians do not show communicative skills but instead have a doctor-centred style that allows for little patient participation [35]. To enhance patients’ experience during physician appointments, it has been suggested to focus on specific approaches tailored to different age groups. For younger patients, these include not rushing the interaction, being engaging and fun, demonstrating a caring demeanor, and ensuring privacy. For older patients, recommended strategies involve active listening, conveying friendliness, fostering long-term relationships, and actively seeking their input [36].

### Strengths and limitations

Strengths of our study were a national population-based sample with a fairly good participation rate. However, our study has some limitations which should be considered when interpreting the findings. Our study may be subject to response bias, but we employed IPW correction method to mitigate this issue [27]. This method has been found suitable for adjusting possible non-response bias among the Finnish population [27]. We adjusted for many factors, however, a possibility of residual confounding still exists. Some variables that were not adjusted for may have influenced the associations we examined and, thus, cause bias to our results. Moreover, the differences in the focus of each appointment types might have affected our results. For example, different appointment types are offered to patients at different stages of care process. A phone appointment is often scheduled to discuss examination results, such as imaging findings from a prior in-person visit. The interaction between the patient and the healthcare professional during the initial contact when the patient first sought care could potentially influence their experiences during follow-up visits. However, the research design carried out here could only take into account patients’ assessments of the most recent appointment. In addition, because we asked patients’ experience of their recent appointment within 12 months, there is a possibility for memory errors which may have affected our results.

The number of appointments conducted by phone call and especially by digital services was rather low compared to in-person appointments which may have affected our results. It is possible that also associations of digital appointments with active participation and responsiveness to needs would have been statistically significant if the number of digital appointments would have been higher. Moreover, one big limitation of our study is that we combined video consultations and chats to same category (digital appointment) which may have affected our results. It would have been more informative if we could have compared also these two appointment types separately with in-person appointments. It is possible that video consultations which include both audio and visual components would have been perceived differently from text-based chat consultations. Thus, it would be good that future studies would examine the differences of all these varying appointment types when teleconsultations have reached wider use. When interpreting our findings, it should be remembered that our findings focus on patients’ perceptions of, for example, time allocation rather than actual time allocation of the appointment. Caution should be remembered when generalising our findings to other countries which may have diverse healthcare system and teleconsultation level, keeping in mind that Finland is amongst the forerunners in the digitalisation of healthcare services.

## Conclusions

Our results show that patients perceive that teleconsultations do not allocate enough time, offer opportunity to ask, give possibility to active participation, and meet patients’ needs at the same amount as in-person appointments. Especially appointments given by phone call differed negatively from in-person visits regarding all these aspects. It is possible that especially vulnerable persons without proper access to digital services might be offered phone call services, and thus be at disadvantaged position. Our findings should be kept in mind when planning and developing teleconsultations. It is important to try to improve patients’ experience of their care given that their insights have been associated, for example, with psychological and symptom distress [37], chronic care management [18], and satisfaction with the medical consultation [38]. It is also important to keep in mind that teleconsultations may be a good option in many cases, but not for all patients or for all situations. Moreover, it would be important that especially physicians and health centers would conduct measures to improve time allocation, patient participation, and responsiveness to patients’ needs.

## Data Availability

The datasets used and analysed during the current study are available from the corresponding author on reasonable request.

## Abbreviations

PREMs: Patient-reported measures
DVV: Digital and Population Data Service Agency
IPW: An Inverse Probability Weighting
